# In Vitro and In Vivo Anticancer Activity of Root Extracts of *Sansevieria liberica* Gerome and Labroy (Agavaceae)

**DOI:** 10.1155/2015/560404

**Published:** 2015-02-24

**Authors:** Abidemi J. Akindele, Zahoor A. Wani, Sadhana Sharma, Girish Mahajan, Naresh K. Satti, Olufunmilayo O. Adeyemi, Dilip M. Mondhe, Ajit K. Saxena

**Affiliations:** ^1^Cancer Pharmacology Division, Indian Institute of Integrative Medicine (Council of Scientific & Industrial Research), Jammu 180001, India; ^2^Department of Pharmacology, Therapeutics & Toxicology (PTT), Faculty of Basic Medical Sciences, College of Medicine, University of Lagos, PMB 12003, Lagos 23401, Nigeria; ^3^Natural Products Chemistry Division, Indian Institute of Integrative Medicine (Council of Scientific & Industrial Research), Jammu 180001, India

## Abstract

*Introduction. Sansevieria liberica* Gerome and Labroy (Agavaceae) is a perennial plant widely distributed in tropical Africa. Preparations of the plant are commonly used across Nigeria for the treatment of inflammatory conditions. Based on the fact that herbal medicine is a strong component of integrative medicine, this study was conducted to evaluate the anticancer activity of root extracts of *Sansevieria liberica. Methods.* Sulforhodamine B (SRB) in vitro cytotoxicity assay, Sarcoma-180 (S-180) ascites and solid tumor, and L1210 lymphoid leukemia in vivo models were used in this study. *Results.* SL-A002 (IC_50_ 23 *µ*g/mL with HeLa), SL-A003 (IC_50_ 22 *µ*g/mL with HCT-116), and SL-A004 (IC_50_ 23 and 18 *µ*g/mL with A549 and THP-1, resp.) demonstrated significant activity in the SRB cytotoxicity assay. Potency was highest with the following pairs of extract : cancer cell line: SL-A002 : HeLa (IC_50_ 23 *µ*g/mL), SL-A003 : HCT-116 (IC_50_ 22 *µ*g/mL), and SL-A004 : THP-1 (IC_50_ 18 *µ*g/mL). SL-A002 demonstrated significant dose-dependent antitumor activity in the Sarcoma-180 (S-180) ascites model with peak effect produced at the dose of 120 mg/kg (i.p.) with inhibition of 89.36% compared to 97.96% for 5-FU (20 mg/kg i.p.). The inhibition of tumor growth by SL-A002 in the S-180 solid tumor model was 47.40% compared to a value of 50.18% for 5-FU. SL-A002 was also significantly active in the L1210 lymphoid leukemia model with 158.33% increase in mean survival time, the same value for 5-FU. *Conclusions.* The hydroethanolic extract of *Sansevieria liberica*, SL-A002, possesses significant anticancer activity to warrant further extensive study to identify, isolate, and characterize the specific bioactive molecules responsible for the observed antitumor activity and the precise mechanism(s) of action.

## 1. Introduction

Cancer is a disease of multicellular organisms [1] characterized by uncontrolled multiplication of subtly modified normal human cells [2]. Cancer is a leading cause of death all over the world and represents a major public health burden [3]. Cancer is the leading cause of death in economically developed countries and the second leading cause of death in developing countries [4] and the burden of cancer is increasing in economically developing countries as a result of population aging and growth as well as, increasingly, an adoption of cancer-associated lifestyle choices including smoking, physical inactivity, and “westernized” diets [5]. It has been estimated that the total number of new cases of cancer will rise from 10 million in year 2000 by approximately 25% in each decade, reaching 24 million new cases per year in the year 2050; the total number of deaths will rise from 6 million in the year 2000 to 10 million in 2020 to over 16 million in the year 2050; in the year 2050, there will be 17 million new cases of cancer in less developed countries, while only 7 million new cases of cancer will occur in the more developed countries [6–8].

Over the years, different approaches have been employed and are still in use, individually or in combination, in the treatment of cancer. These include chemotherapy, radiotherapy, surgery, and immunotherapy. While surgery and radiation therapy are used to treat localized cancers, chemotherapy is used to treat cancer cells that have metastasized to other parts of the body because they travel throughout the body in the bloodstream [1]. The battery of available drugs for systemic treatment of cancer encompasses alkylating agents, antimetabolites, antibiotics, and hormones [9, 10]. Chemotherapeutic agents are cytotoxic and, apart from affecting tumor cells, these active principles also deleteriously impact on rapidly proliferating normal cells, including those localized in the gastrointestinal tract, hair, and bone marrow, thus eliciting gastrointestinal side effects like nausea and vomiting, alopecia, and myelosuppression. According to Rao et al. [1], the efficacy of cancer drugs is often limited by their insolubility and instability, the low rate at which the tissue absorbs them, and tumor's drug resistance. Antitumor drugs have also been associated with development of secondary malignancy. All of the drawbacks presently associated with available chemotherapeutic agents are impetus for the search for newer, more efficacious, and better tolerated drugs. Natural products, especially the plant kingdom, offer an inexhaustible reservoir for investigation.

Plants have a long history of use in the treatment of cancer [11, 12] and the interest in nature as a source of potential chemotherapeutic agents continues [8]. The present day research and development tailored towards the discovery of new antiproliferative agents from natural products have been buoyed by improvement in the science and technology of anticancer drug discovery.

*Sansevieria liberica *Gerome and Labroy (Agavaceae) is a perennial plant with thick woody rhizomes widely distributed in the tropical, subtropical, and temperate zones of the world, commonly located in shady places near streams and rocky parts. The plant is commonly called “mother-in-law tongue,” “African bowstring,” and “Leopard lily.” Local names in Nigeria include “Mooda” (Hausa; north), “Ebube-agu” (Igbo; south-east), “Okonno” (Efik; south-south), and “Oja ikoko” (Yoruba; south-west). Preparations of the plant are used in the treatment of ear and eye infections, inflammation (leaf juice); tooth ache (fruit juice together with fluid from snails); fever, headache, and cold (fume from burning leaves inhaled); cough, pain, inflammation, infections, convulsion, diarrhoea, and as stimulating tonic (root decoction) [13]. Burkill [14] reported the use of the leaf and root preparations of the plant in the treatment of haemorrhoids; ear and eye troubles; pain; smallpox, chicken-pox, and measles; venereal diseases; malnutrition; paralysis, epilepsy, convulsions, and spasm; pulmonary troubles; and as vermifuge. Bero et al. [15] also reported the use of the plant as remedy for parasitic infections. The antidiarrhoeal [16], hepatoprotective [17], CNS depressant and anticonvulsant [18, 19], analgesic [19], anti-inflammatory [20], in vitro antitrypanosomal, and antileishmanial and antiplasmodial [15, 21] activities of extracts of the plant had been reported.

Long-standing inflammation secondary to chronic infection or irritation predisposes to cancer, and cancer and inflammation are related by epidemiology, histopathology, inflammatory profiles, and the efficacy of anti-inflammatory drugs in prophylaxis [22]. Based on the fact that numerous anti-inflammatory agents including those identified from natural sources have been shown to exhibit chemopreventive activities [23, 24], Aggarwal et al. [23] reported that such anti-inflammatory agents can be used not only for prevention but also for therapy of cancer.

Going by its use for the treatment of chronic inflammatory conditions [25], this study was designed to evaluate the anticancer activity of root extracts of* Sansevieria liberica* using a combination of in vitro and in vivo models.

## 2. Materials and Methods

### 2.1. Plant Material

*Sansevieria liberica* roots were obtained from Mushin, Lagos State, Nigeria. The plant material was identified and authenticated at the Forestry Research Institute of Nigeria (FRIN), Ibadan, Nigeria, by Mr. T. K. Odewo (Senior Superintendent), and the Department of Botany, Faculty of Science, University of Lagos, Lagos, Nigeria, by Prof. J. D. Olowokudejo. A voucher specimen (FHI 107621) was deposited in the herbarium of FRIN.

### 2.2. Extraction

The fresh roots of* Sansevieria liberica* were chopped into small pieces and air-dried at room temperature over several weeks until a constant weight was obtained. The dried pieces were powdered and weighed to obtain 4 portions of 100 g each. Each of 3 portions of the milled plant was macerated with alcohol (95% ethanol: A001), hydroalcohol (ethanol and water, 1 : 1; A002), and distilled water (A003), respectively, using 1500 mL of the individual solvents. Maceration was done for 3 h with mechanical stirring (Heidolph RZR 2051 Control) with the speed set at 400 rpm. After maceration with stirring for 3 h, filtration was carried out using Whatman filter paper (150 mm). Residues were remacerated in the respective solvents also for 3 h with mechanical stirring (×2) to achieve exhaustive extraction.

The combined filtrate of the alcoholic extracts was concentrated using Heidolph Rotavapor (LABORATA 4000) with the speed set at 120 rpm and temperature at 40°C. The concentrated extract was removed from the round bottom flask with methanol and poured into weighed beakers. The alcoholic solvent was allowed to evaporate. The alcoholic extract container was designated as A001 and subsequently put into a desiccator. The combined filtrate of the hydroalcoholic extracts was evaporated to dryness with Rotavapor. The dried solid extract was scrapped from the round bottom flask into a weighed plastic container and designated as A002 with the precise extract weight indicated on the container. This container was also subsequently put into a desiccator. The combined filtrate of the aqueous extracts of the plant was lyophilized, weighed, and put into a container designated as A003.

The fourth portion of 100 g of the powdered plant material was put into a 2000 mL separating funnel with the bottom lined with cotton wool and 1 L of dichloromethane : methanol (DCM : MeOH; 70 : 30) was put into the separating funnel. Twenty-four hours after, the solvent mixture was drained and the extraction liquid was filtered using Whatman filter paper (150 mm). On the second and third days, 600 mL of the DCM : MeOH solvent mixture was added to the separating funnel, allowed to drain for 24 h, and filtered. The combined DCM : MeOH extract was concentrated with Rotavapor without vacuum and removed from the round bottom flask with methanol and poured into a weighed beaker. The solvent was allowed to evaporate; the container was designated as A004 and subsequently put into a desiccator. The yield (%) of the extracts was obtained to be 19.49 (A001), 50.19 (A002), 46.37 (A003), and 17.27 (A004).

### 2.3. Chemicals

These were RPMI-1640, minimum essential medium (MEM), fetal calf serum, trypsin, trypan blue, ethanol, penicillin, streptomycin, gentamycin, dimethyl sulfoxide (DMSO), sulforhodamine, mitomycin-C, paclitaxel, and 5-fluorouracil (SIGMA Chemical Co., USA); phosphate buffer saline (PBS, MERCK, Germany); trichloroacetic acid (TCA), distilled water, sodium hydroxide, Tris-EDTA buffer, Tris buffer (Hi-Media); acetic acid, sodium bicarbonate, hydrochloric acid (RANKEM, New Delhi, India), isopropanol (SISCO, Mumbai, India), and Tris-acetate-EDTA buffer. All other chemicals used in this study were of analytical grade and were purchased locally.

### 2.4. Cell Lines and Cell Cultures

A549 (lung), HCT-116 (colon), PC3 (prostate), A431 (skin), HeLa (cervix), and THP-1 (leukemia) human cancer cell lines were obtained from the National Cancer Institute, Frederick, USA. The cells were grown and maintained in appropriate medium, pH 7.4, supplemented with 10% fetal calf serum, glutamine (2 mM), penicillin (100 units/mL), and streptomycin (100 *μ*g/mL). The cell cultures were grown in a carbon dioxide incubator (Heraeus, GmbH, Germany) at 37°C with 90% humidity and 5% CO_2_ [26, 27].

### 2.5. Animals

Inbred BALB/c, outbred Swiss albino, DBA/2, and CDF1 mice used in this study were obtained from different laboratories. The animals were maintained at 23 ± 2°C with 20–25 complete air changes with 100% fresh air, with relative humidity maintained at 50–60%. The mice were housed in transparent polycarbonate filter top cages in animal isolator cabins and were fed with pelleted feed (M/s Ashirwad Industries, Chandigarh, India) and autoclaved water* ad libitum*. Mice selected for experiments were of the same sex and strain, healthy, and free from any disease. These were in the weight range of 18–23 g (about 2 months of age). The experimental procedures employed in this study were approved by the Institutional Animal Ethics Committee, Indian Institute of Integrative Medicine, Jammu, India.

### 2.6. In Vitro Cytotoxicity against Human Cancer Cell Lines

The in vitro cytotoxicity of the extracts of* Sansevieria liberica* was determined by semiautomated assay using sulforhodamine-B (SRB) [26–28]. The human cancer cell lines were grown in tissue culture flasks at 37°C in an atmosphere of 5% CO_2_ and 90% relative humidity in complete growth medium. Flasks with subconfluent stage of growth were selected and cells were harvested by treatment with trypsin-EDTA. The number of cells/mL of suspension was counted using haemocytometer. The cell density was adjusted to 10,000 cells/100 *μ*L, or as appropriate for each cell line, in the cell suspension. One hundred *μ*L of cell suspension was added to each well of 96-well plates with the help of handy-step. The plates were incubated at 37°C in an atmosphere of 5% CO_2_ and 90% relative humidity for 24 h. Thereafter, 100 *μ*L of working solution of each test material was added to the wells of the 96-well plates. The stock solutions of the extracts (20 mg/mL) were prepared in DMSO and serially diluted with complete growth medium such that 100 *μ*L of working solutions of each extract gave concentrations of 10, 30, and 100 *μ*g/mL (final DMSO concentration was 0.5% highest to 0.001% lowest) added to the 96-well cell culture plates. The 96-well cell culture plates contained appropriately seeded cells (e.g., 8000 cells/100 *μ*L for HCT-116 and A431; 10000 cells/100 *μ*L for HeLa) and all vehicle controls contained the same concentration of DMSO.

The plates were incubated for 48 h at 37°C in an atmosphere of 5% CO_2_ and 90% relative humidity. Thereafter, 50 *μ*L of chilled 50% TCA was gently added to each well of the plates, making a final concentration of 10%. The plates were incubated at 4°C for 1 h to fix the cells attached to the bottom of the wells. The plates were then washed 5-6 times with distilled water and thereafter air-dried. To each well, 100 *μ*L of SRB dye (0.4% wt/vol in 1% acetic acid) was added and left at room temperature for 30 min. Thereafter, the plates were washed with 1% acetic acid. The plates were again air-dried and 100 *μ*L of Tris buffer (10 mM; pH 10.5) was added to each well. The plates were shaken gently for 10–15 min. on a mechanical shaker. Blank wells contained medium but no cells and the control wells contained cells but no test samples. The optical density (OD) of the plate wells was recorded with a microplate reader at 540 nm and data were maintained. Growth inhibition was calculated as the percent survival of treated cells over control cells × 100 (*T*/*C*%):

(1) %Growth  inhibition =100−ODtest  sample−ODblankODcontrol−ODblank  ×100.

### 2.7. In Vivo Anticancer Activity Evaluation

#### 2.7.1. Sarcoma-180 (S-180) Ascites Model

This was carried out according to the methods described by Monks et al. [29] and Chashoo et al. [30]. S-180 cells were harvested from the peritoneal cavity of Swiss albino mice, used for propagation, harbouring 8–10-day-old ascitic tumor. On day 0, 1 × 10^7^ cells/animal was injected i.p. into the peritoneal cavity of BALB/c mice of the same sex. The tumor infected animals were then randomized and divided into different groups based on the treatment schedule, including one control (normal saline) group and one standard drug (5-FU) group. From days 1 to 9, the different treatment groups were administered i.p. SL-A001, A003, A004 (100 mg/kg), and A002 (80 mg/kg), 5-FU (20 mg/kg), and normal saline (0.2 mL/mouse). On day 12, all the animals were sacrificed under diethyl ether anesthesia and the ascitic fluid was collected from the peritoneal cavity of each mouse for the evaluation of tumor weight, volume, and cell number. The percent inhibition of tumor was calculated; thus

(2) Average  number  of  cells  in  controlsAs−1 −Average  number  of  cells  in  treated  animals ·Average  number  of  cells  in  controls−1×100.

Based on the highest value of tumor growth inhibition and least mortality, SL-A002 was subjected to a graded dose (80, 100, and 120 mg/kg i.p.) evaluation in this model.

#### 2.7.2. Sarcoma-180 (S-180) Solid Tumor Model

This evaluation followed the same procedure outlined in the ascites model except that tumor cells (1 × 10^7^ cells/animal) were injected i.m. into the right thigh of BALB/c mice of the same sex on day 0. Based on its effectiveness in the ascites model, SL-A002 was evaluated in this model at the dose of 100 mg/kg i.p. Normal saline (0.2 mL/mouse) and 5-FU (20 mg/kg) given i.p. served as control and standard drug, respectively. On day 13, the longest and shortest diameters of tumors were measured with a vernier caliper and the tumor volume was determined according to established procedure [31, 32]:

(3) $$\begin{array}{c} \text{Tumor}\;\text{volume}\left({\text{mm}}^{3}\right)=\frac{\left(L\times W^{2}\right)}{2}. \end{array}$$

Percent inhibition of tumor:

(4) Average  tumor  volume  of  controlsAs−1 −Average  tumor  volume  of  treated  animals ·Average  tumor  volume  of  controls−1×100.

#### 2.7.3. L1210 Lymphoid Leukemia Model

L1210 lymphocytic leukemia cells were harvested from the peritoneal cavity of DBA/2 mice used for propagation, harboring 7-day tumor cells. On day 0, 2.5 × 10^6^ cells were injected i.p. into CDF1 mice of the same sex. From days 1 to 9, the different treatment groups were administered i.p. SL-A002 (100 mg/kg), 5-FU (20 mg/kg), and normal saline (0.2 mL/mouse). The animals were observed for mortality and the mean survival time (MST) was calculated; thus

(5) $$\begin{array}{c} \frac{\sum S+6S_{5}-19\text{NT}}{S_{5}-\text{NT}}, \end{array}$$

where *S*_5_ is number of survivors on day 5, ∑*S* is sum of daily survivors from day 6 to day 18, and NT is number of no takes (survivors beyond day 18):

(6) $$\begin{array}{c} \%\frac{T}{C}=\left(\text{MS}{\text{T}}_{\text{Treatment}}÷\text{MS}{\text{T}}_{\text{Control}}\right)\times 100, \end{array}$$

where *T* is mean survival time (MST, days) of the drug treated mice, *C* is mean survival time (MST, days) of untreated control animals, *T*/*C*% < 125% is toxic/inactive, and *T*/*C*% > 125% is significant antileukemic effect [33].

### 2.8. Statistical Analysis

The results obtained in this study are displayed as mean ± SEM. Data analysis was done using one-way ANOVA followed by Dunnett's multiple comparison test (GraphPad Prism 5, GraphPad Software Inc., La Jolla, CA, USA). Values were considered significant at *P* < 0.05.

## 3. Results

### 3.1. In Vitro Cytotoxic Activity

The results of the in vitro cytotoxicity using SRB assay are presented herein based on the recommendation of the National Cancer Institute (NCI, USA) that 30 *μ*g/mL is the upper IC_50_ limit considered promising for purification of a crude extract [34].

**Figure 1:**
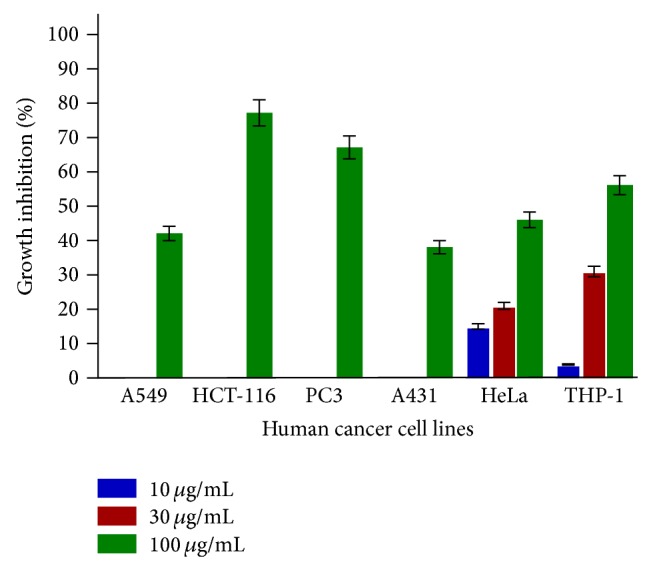
In vitro cytotoxic activity of SL-A001 against various human cancer cells lines in the SRB assay. Estimated IC_50_ values are >100, 75, 82, >100, >100, and 82 *μ*g/mL for A549, HCT-116, PC3, A431, HeLa, and THP-1, respectively.

**Figure 2:**
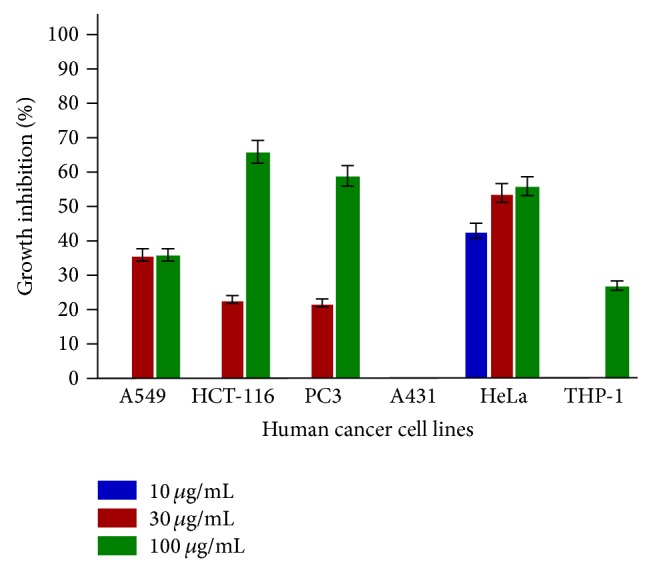
In vitro cytotoxic activity of SL-A002 against various human cancer cells lines in the SRB assay. Estimated IC_50_ values are >100, 73, 83, NA, 23, and >100 *μ*g/mL for A549, HCT-116, PC3, A431, HeLa, and THP-1, respectively. NA implies not active.

**Figure 3:**
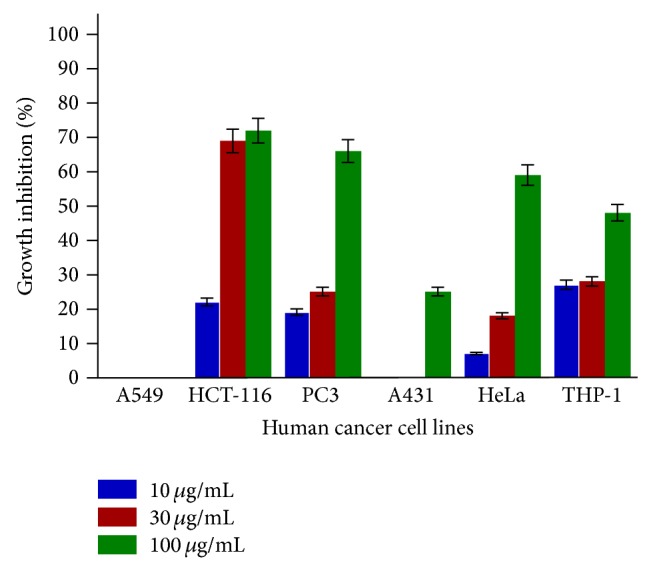
In vitro cytotoxic activity of SL-A003 against various human cancer cells lines in the SRB assay. Estimated IC_50_ values are NA, 22, 72, >100, 85, and >100 *μ*g/mL for A549, HCT-116, PC3, A431, HeLa, and THP-1, respectively. NA implies not active.

**Figure 4:**
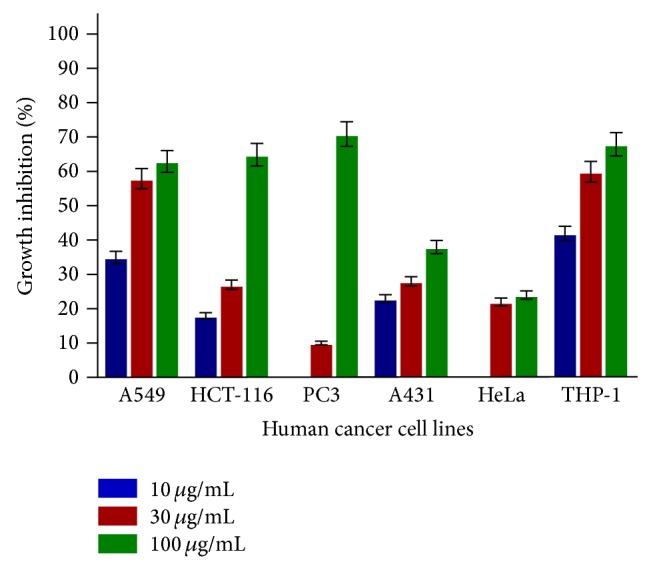
In vitro cytotoxic activity of SL-A004 against various human cancer cells lines in the SRB assay. Estimated IC_50_ values are 23, 72, 75, >100, >100, and 18 *μ*g/mL for A549, HCT-116, PC3, A431, HeLa, and THP-1, respectively.

SL-A001 did not produce significant effect on all the human cancer cell lines used in this study (Figure 1). SL-A002 and SL-A003 showed significant activity on HeLa and HCT-116 human cancer cell lines with IC_50_ values of 23 and 22 *μ*g/mL, respectively (Figures 2 and 3). In respect of SL-A004, as shown in Figure 4, significant cytotoxic activity was elicited on A549 and THP-1 human cancer cell lines with IC_50_ values of 23 and 18 *μ*g/mL, respectively.

### 3.2. In Vivo Anticancer Activity of SL Extracts against Sarcoma-180 Ascites in Balb/c Mice

**Figure 5:**
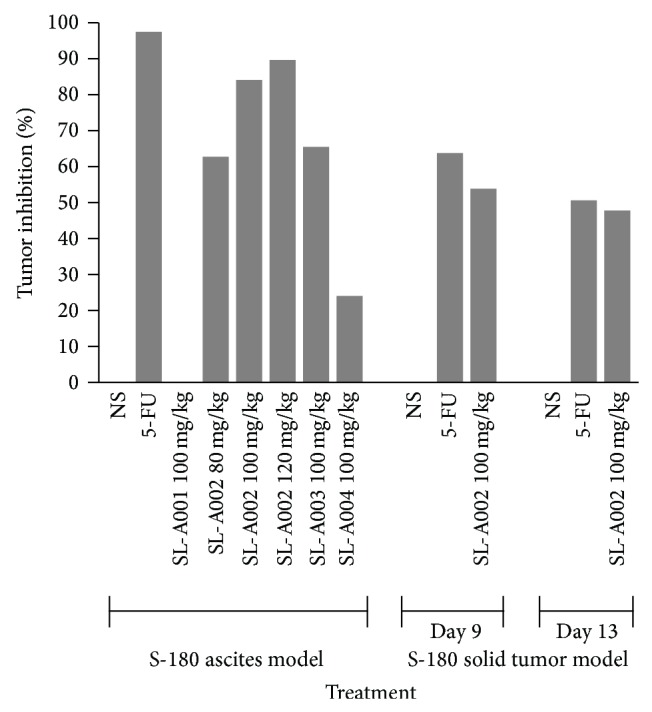
Graphical representation of tumor inhibition values in the S-180 ascites and solid tumor models.

The initial screening results for the effects of SL extracts on Sarcoma-180 ascites model in mice are shown in Table 1. SL-A001 (100 mg/kg), SL-A002 (80 mg/kg), SL-A003, and SL-A004 (100 mg/kg) produced tumor growth inhibition values, based on the number of tumor cells, of 0, 62.34, 65.15, and 23.54%, with mortality values of 0, 0, 42.86, and 14.29%, respectively. The inhibitory effects were significant (*P* < 0.01) compared to control in respect of SL-A002 and SL-A003, but these effects were significantly lower (*P* < 0.05, 0.01) compared to 5-FU (97.19%). The effect of SL-A004 was also significantly lower (*P* < 0.001) compared to 5-FU. SL-A002 also caused significant (*P* < 0.01, 0.001) inhibition of tumor growth at doses of 100 and 120 mg/kg with values of 83.80 and 89.36%, respectively, with no mortality, compared to 5-FU (97.96%). This shows dose-dependency in its antitumor effect considering the doses of 80, 100, and 120 mg/kg used in this study (Table 2). A graphical representation of tumor inhibition values in this model is presented in Figure 5.

### 3.3. In Vivo Anticancer Activity of SL-A002 Extract against Sarcoma-180 Solid Tumor in Balb/c Mice

SL-A002 (100 mg/kg) produced significant inhibition in tumor growth on days 9 and 13 with values of 53.42 and 47.40%, respectively. These effects were comparable and not significantly different (*P* > 0.05) from those elicited by 5-FU (63.39 and 53.42%, resp.). SL-A002 also caused significant reductions (*P* < 0.01, 0.001) in body weight on days 5, 9, and 13 (Table 3). A graphical representation of tumor inhibition values in this model is presented in Figure 5.

### 3.4. Effects of SL-A002 Extract against L1210 Lymphoid Leukemia in CDF1 Mice

The effect of SL-A002 (100 mg/kg) on L1210 lymphoid leukemia model is shown in Table 4. SL-A002 increased the mean survival time from 12 days to 19 days corresponding to 158.33% increase in mean survival time. This value was the same as for 5-FU.

## 4. Discussion

Cancer therapy in the form of surgery or radiotherapy is effective when the disease is early detected but many cancers are still diagnosed when cells from a primary tumor have already metastasized to other parts of the body and the main form of treatment at this point is chemotherapy [35]. Chemotherapy entails delivering drugs systemically so that they can reach and kill the tumor cells, but most of these drugs cause severe side effects in patients and, therefore, need to be used at suboptimal levels. According to Jemal et al. [36], the low efficacy of chemotherapy in patients with advanced cancers is reflected in the low 5-year survival rates observed in these patients and the low efficacy of cancer therapy for the treatment of patients with metastasis makes the development of novel chemotherapeutic agents necessary. Denny and Wansbrough [37] reported that a major challenge is to design new drugs that will be more selective for cancer cells and thus have lesser side effects.

Integrative medicine with the approach of combining conventional western medicine with alternative or complementary treatments, such as herbal medicine, acupuncture, massages, biofeedback, yoga, and stress reduction techniques [38], is being used to complement orthodox medicines and treatment approaches in the management of cancer patients. According to Lammersfeld [39] patients may turn to integrative therapies when the disease they are battling does not respond to traditional medical therapies and/or to help reduce symptoms while improving overall well-being, and among cancer patients more than half use some kind of integrative therapy, according to a 2012 meta-analysis in integrative cancer therapies.

Plants have served as a rich source of therapeutic agents for many centuries, being used themselves or as the basis for synthetic drugs [40], and despite the great developments in organic synthesis, 55% of recent chemotherapeutic drugs are derived from or based upon natural products [41]. The use of plants as food and in folk and traditional medicine has made these natural resources one of the main agents in the research and development of cancer chemopreventive drugs [42, 43]. According to Rates [44] and Jemal et al. [45], the interest in alternative therapies using natural products is increasing, especially those derived from plants, due to the increasingly high number of cancer cases worldwide. In order to look for new sources of therapeutic anticancer agents, many plant extracts and active principles have been studied in in vitro and in vivo cancer models, and the correlation of both studies became one of the key steps for the success of this type of research [40, 41].

In this study, the hydroethanol extract of the root of* Sansevieria liberica* (SL-A002) was significantly active (IC_50_ ≤ 30 *μ*g/mL [34]) against HeLa cancer cell line, the aqueous extract (SL-A003) against HCT-116, and the DCM : MeOH extract (SL-A004) against THP-1 and A549 human cancer cell lines. Only SL-A002 showed significant activity in the Sarcoma-180 ascites model with peak tumor growth inhibition of 89.36% produced at the dose of 120 mg/kg relative to 97.96% for 5-FU at 20 mg/kg. The hydroethanol extract of* Sansevieria liberica* was subsequently found to be active in the Sarcoma-180 solid tumor model eliciting 47.40% tumor growth inhibition at the dose of 100 mg/kg compared to 50.18% for 5-FU at the dose of 20 mg/kg. This extract was also found to be significantly active in the L1210 lymphoid leukemia model with T/C value of 158.33% at the dose of 100 mg/kg, the same value for 5-FU (20 mg/kg).

Prior to this study, no report of anticancer activity of* Sansevieria liberica* was seen from extensive literature search. However, other species have been investigated and reported [46–50].

## 5. Conclusion

Based on the results obtained in this study, in which the SRB in vitro cytotoxicity assay, the Sarcoma-180 ascites, Sarcoma-180 solid, and L1210 lymphoid leukemia in vivo models were used, it can be concluded that the hydroethanolic root extract of* Sansevieria liberica*, SL-A002, possesses significant anticancer activity to warrant further extensive study. Further research works are in view to identify, isolate, and characterize the specific bioactive molecules responsible for the observed antitumor activity and their precise mechanism(s) of action.

## Figures and Tables

**Table 1:** Effects of SL extracts against Sarcoma-180 ascites in BALB/c mice.

Treatments	Dose (mg/kg)	Body weight (g) Day 1	Body weight (g) Day 5	Body weight (g) Day 9	Body weight (g) Day 12	Weight of tumor (g)	Volume of ascitic fluid (mL)	Number of tumor cells (×10^7^)	Tumor growth inhibition (%)	Mortality (%)
Normal saline	(0.2 mL/mouse)	21.09 ± 0.56	22.91 ± 0.71	25.18 ± 0.97	24.63 ± 1.49	7.28 ± 0.79	7.47 ± 0.80	178.07 ± 24.98	0	0
5-FU	20	20.00 ± 0.53	20.57 ± 0.69^a^	19.43 ± 1.27^b^	19.83 ± 1.45^a^	0.58 ± 0.37^c^	0.53 ± 0.34^c^	5.00 ± 3.42^c^	97.19	0
SL-A001	100	19.57 ± 0.57^a^	21.43 ± 0.72	23.14 ± 0.70^*α*^	24.00 ± 1.02^*α*^	8.34 ± 0.45^*γ*^	8.81 ± 0.48^*γ*^	214.25 ± 27.50^*γ*^	0	0
SL-A002	80	19.57 ± 0.57^a^	18.57 ± 0.75^c,*α*^	18.17 ± 0.70^c^	17.80 ± 0.97^b^	3.16 ± 0.54^c,*β*^	3.10 ± 0.62^c,*β*^	67.06 ± 12.54^b,*β*^	62.34	0
SL-A003	100	19.86 ± 0.55	17.71 ± 0.29^c,*β*^	16.80 ± 0.37^c,*α*^	16.25 ± 0.48^c,*α*^	2.13 ± 0.75^c^	2.13 ± 0.75^c^	62.06 ± 20.87^b,*α*^	65.15	42.86
SL-A004	100	20.00 ± 0.53	20.71 ± 0.57^a^	22.67 ± 0.67^a,*α*^	25.00 ± 1.03^*β*^	8.55 ± 0.61^*γ*^	8.63 ± 0.54^*γ*^	136.16 ± 8.09^*γ*^	23.54	14.29

Values are mean ± SEM (*n* = 7, 11 for control). ^a^ *P* < 0.05, ^b^ *P* < 0.01, and ^c^ *P* < 0.001 versus normal saline; ^*α*^ *P* < 0.05, ^*β*^ *P* < 0.01, and ^*γ*^ *P* < 0.001 versus 5-FU.

**Table 2:** Effects of SL-A002 extract (100 and 120 mg/kg) against Sarcoma-180 ascites in BALB/c mice.

Treatments	Dose (mg/kg)	Body weight (g) Day 1	Body weight (g) Day 5	Body weight (g) Day 9	Body weight (g) Day 12	Weight of tumor (g)	Volume of ascitic fluid (mL)	Number of tumor cells (×10^7^)	Tumor growth inhibition (%)	Mortality (%)
Normal saline	(0.2 mL/mouse)	22.20 ± 0.49	24.00 ± 0.55	26.80 ± 0.49	29.60 ± 0.93	8.74 ± 0.52	8.92 ± 0.51	686.79 ± 54.40	0	0
5-FU	20	21.80 ± 0.58	21.20 ± 0.92^a^	19.00 ± 1.26^c^	18.00 ± 1.82^c^	0.52 ± 0.24^c^	0.50 ± 0.22^c^	14.00 ± 5.74^c^	97.96	0
SL-A002	100	21.80 ± 0.58	21.60 ± 0.51^b^	21.20 ± 0.37^c^	22.60 ± 0.81^c,*α*^	3.32 ± 0.41^c,*γ*^	3.18 ± 0.44^c,*γ*^	111.25 ± 18.93^c,*β*^	83.80	0
	120	21.80 ± 0.58	20.40 ± 0.87^b^	19.60 ± 0.98^c^	20.00 ± 1.82^b^	2.85 ± 1.15^b^	2.84 ± 1.10^b^	73.09 ± 18.18^c,*α*^	89.36	0

Values are mean ± SEM (*n* = 5). ^a^ *P* < 0.05, ^b^ *P* < 0.01, and ^c^ *P* < 0.001 versus normal saline; ^*α*^ *P* < 0.05, ^*β*^ *P* < 0.01, and ^*γ*^ *P* < 0.001 versus 5-FU.

**Table 3:** Effects of SL-A002 extract against Sarcoma-180 solid tumor in BALB/c mice.

Treatments	Dose (mg/kg)	Body weight (g) Day 1	Body weight (g) Day 5	Body weight (g) Day 9	Body weight (g) Day 13	Tumor volume (mm^3^) Day 9	Inhibition (%) Day 9	Tumor volume (mm^3^) Day 13	Inhibition (%) Day 13
Normal saline	(0.2 mL/mouse)	21.80 ± 0.58	23.00 ± 0.55	23.80 ± 0.58	24.00 ± 0.32	1848.50 ± 108.31	0.00	2283.50 ± 136.58	0.00
5-FU	20	21.80 ± 0.58	20.60 ± 1.17	18.80 ± 2.08^a^	19.80 ± 2.15	676.80 ± 188.15^c^	63.39	1137.60 ± 247.45^b^	50.18
SL-A002	100	21.20 ± 0.66	19.20 ± 0.80^b^	19.60 ± 0.87^b^	19.80 ± 0.73^b^	861.10 ± 157.60^c^	53.42	1201.20 ± 232.26^b^	47.40

Values are mean ± SEM (*n* = 5). ^a^ *P* < 0.05, ^b^ *P* < 0.01, and ^c^ *P* < 0.001 versus normal saline.

**Table 4:** Effect of SL-A002 extract against L1210 lymphoid leukemia in CDF1 mice.

Treatments	Dose (mg/kg)	Mean survival time	Increase in mean survival time (%)	Inference
Normal saline	(0.2 mL/mouse)	12	—	—
5-FU	20	19	158.33	Significant activity
SL-A002	100	19	158.33	Significant activity

*T*/*C*% < 125% = toxic/inactive.

*T*/*C*% > 125% = significant antileukemic effect.

*n* = 6,10 for control.
